# Dataset of the associations of aldosterone to renin ratio with MR-proANP and MR-proADM

**DOI:** 10.1016/j.dib.2016.08.008

**Published:** 2016-08-09

**Authors:** Cornelia Then, Marietta Rottenkolber, Andreas Lechner, Christa Meisinger, Margit Heier, Wolfgang Koenig, Annette Peters, Wolfgang Rathmann, Martin Bidlingmaier, Martin Reincke, Jochen Seissler

**Affiliations:** a Medizinische Klinik und Poliklinik IV, Klinikum der Ludwig-Maximilians-Universität, Munich, Germany; b Clinical Cooperation Group Diabetes, Ludwig-Maximilians-Universität München and Helmholtz Zentrum München, Munich, Germany; c Institute of Epidemiology II, Helmholtz Zentrum München – German Research Center for Environmental Health (GmbH), Neuherberg, Germany; d Department of Internal Medicine II – Cardiology, University of Ulm, Medical Centre, Ulm, Germany; e Deutsches Herzzentrum München, Technische Universität München, Munich, Germany; f DZHK (German Centre for Cardiovascular Research), partner site Munich Heart Alliance, Munich, Germany; g Research Unit of Molecular Epidemiology, German Research Center for Environmental Health, Neuherberg, Germany; h German Diabetes Center, Leibniz Institute at Heinrich Heine University Düsseldorf, Institute of Biometrics and Epidemiology, Düsseldorf, Germany

**Keywords:** Aldosterone to renin ratio, MR-proANP, MR-proADM, Diabetes

## Abstract

This article contains data related to the research article entitled “Altered relation of the renin-aldosterone system and vasoactive peptides in type 2 diabetes: the KORA F4 study” (Then et al., 2016) [1] and describes the association of the aldosterone to renin ratio with midregional-pro atrial natriuretic peptide (MR-proANP) and midregional-pro adrenomedullin (MR-proADM) in 1261 participants from the KORA F4 cohort.

**Specifications Table**

**Table:** 

Subject area	*Diabetes*
More specific subject area	*Cardiovascular disease in diabetes*
Type of data	*Tables*
How data was acquired	*Cohort study*
Data format	*Analyzed*
Experimental factors	*Plasma samples were obtained from 1261 participants of the population-based KORA F4 cohort after an overnight fast.*
Experimental features	*Plasma MR-proANP, MR-proADM, renin and aldosterone were measured. Linear regression models were used to assess the association between MR-proANP/MR-proADM and aldosterone-renin-ratio.*
Data source location	*Cooperative Health Research in the Region of Augsburg, Southern Germany*
Data accessibility	*The data is with this article*

**Value of the data**

• The aldosterone to renin ratio was only associated with MR-proANP and MR-proADM in subjects without diabetes or prediabetes.
• The current data may be helpful for the planning of further clinical and/or preclinical research aiming to work out the differences in vasoactive hormones in subjects with and without diabetes.
• Vasoactive prohormones, including MR-proANP and MR-proADM, are interesting candidates for future research focusing on the pathophysiological links between diabetes and cardiovascular disease.

## 1 Data

Two Tables are presented showing the associations of vasoactive prohormones with the aldosterone to renin ratio (ARR) in subjects with and without diabetes type 2 or prediabetes in a population-based cohort. Table 1 displays the association of the ARR with MR-proANP and Table 2 shows the relation of ARR and MR-proADM.

## 2 Experimental design, materials and methods

Recruitment of study participants, laboratory measurements and statistical analyses are described in detail elsewhere [1]. Briefly, the population-based KORA (Cooperative Health Research in the Region of Augsburg, southern Germany) F4 cohort includes 3080 participants recruited between 2006 and 2008. From this cohort, a sample of 1596 subjects was randomly selected for plasma MR-proANP and MR-proADM measurements. All variables required for the currently described analyses were available in 1261 study participants. ARR was calculated by dividing plasma aldosterone levels (ng/l) by plasma renin levels (ng/l).

## Figures and Tables

**Table 1:** Association of ARR (logarithmized) and MR-proANP (linear regression). Bold: *p*<0.05.

	**MR-proANP Q4 vs Q1-Q3**	***p-value***	**MR-proANP Q4 vs Q1**	***p-value***	**MR-proANP metric**	***p-value***
	**Regression coefficient (95% CI)**		**Regression coefficient (95% CI)**		**Regression coefficient (95% CI)**	
**No adjustment**						
All subjects	0.030 (−0.119 to 0.179)	0.694	**0.214 (0.020–0.408)**	**0.031**	0.00071 (−0.00047 to 0.00189)	0.237
Prediabetes	0.299 (−0.081 to 0.679)	0.123	0.143 (−0.31–0.597)	0.532	0.00284 (−0.00008 to 0.00575)	0.056
Diabetes	−0.344 (−0.88 to 0.192)	0.206	−0.494 (−1.193 to 0.204)	0.163	−0.00222 (−0.00578 to 0.00134)	0.219
No diabetes/prediabetes	0.163 (−0.005 to 0.331)	0.057	**0.407 (0.184–0.629)**	**<0.001**	0.00131 (−0.00009 to 0.00271)	0.066
**Adjusted for age and sex**						
All subjects	0.039 (−0.134 to 0.211)	0.661	0.289 (−0.014 to 0.593)	0.062	0.00076 (−0.00059 to 0.00211)	0.267
Prediabetes	0.322 (−0.099 to 0.744)	0.133	−0.111 (−0.754 to 0.532)	0.732	0.00317 (−0.00013 to 0.00648)	0.060
Diabetes	−0.425 (−1.015 to 0.164)	0.156	−0.589 (−1.445 to 0.266)	0.173	−0.00274 (−0.00669 to 0.00121)	0.173
No diabetes/prediabetes	0.111 (−0.088 to 0.309)	0.274	**0.477 (0.133–0.821)**	**0.007**	0.00057 (−0.00103 to 0.00218)	0.484
**Adjusted for age, sex and BMI**						
All subjects	0.025 (−0.146 to 0.197)	0.771	0.2851 (−0.012 to 0.582)	0.060	0.00066 (−0.00068 to 0.00200)	0.336
Prediabetes	0.301 (−0.120 to 0.722)	0.160	−0.144 (−0.790 to 0.502)	0.660	0.00288 (−0.00044 to 0.00619)	0.090
Diabetes	−0.423 (−1.022 to 0.177)	0.165	−0.504 (−1.416 to 0.408)	0.273	−0.00271 (−0.0067 to 0.00128)	0.182
No diabetes/prediabetes	0.098 (−0.100 to 0.296)	0.332	**0.444 (0.106–0.781)**	**0.010**	0.00057 (−0.00103 to 0.00217)	0.488
**Multivariable adjustment**^a^						
All subjects	**0.178 (0.003–0.354)**	**0.046**	**0.375 (0.068–0.681)**	**0.017**	**0.00230 (0.00088–0.00372)**	**0.002**
Prediabetes	**0.528 (0.110–0.946)**	**0.013**	0.035 (−0.686–0.756)	0.924	**0.00661 (0.00316–0.01006)**	**<0.001**
Diabetes	−0.104 (−0.775 to 0.567)	0.759	−0.152 (−1.319–1.014)	0.794	0.00032 (−0.00422 to 0.00486)	0.890
No diabetes/prediabetes	0.196 (−0.011 to 0.403)	0.063	**0.532 (0.180–0.884)**	**0.003**	0.00142 (−0.0003 to 0.00313)	0.106

a Adjustment for age, sex, BMI, hypertension (yes/no), HDL cholesterol (continuous), LDL cholesterol (continuous), triglycerides (continuous), former myocardial infarction or stroke, smoking behaviour (active/former/never), alcohol consumption (never/moderate/high), physical activity (high/low), hsCRP, eGFR, statins, NSAID, beta-blockers, angiotensin converting enzyme inhibitors and angiotensin 1 receptor antagonists.

**Table 2:** Association of ARR (logarithmized) and MR-proADM (linear regression). Bold: *p*<0.05.

	**MR-proADM Q4 vs Q1-Q3**	***p-value***	**MR-proADM Q4 vs Q1**	***p-value***	**MR-proADM metric**	***p-value***
	**Regression coefficient (95% CI)**		**Regression coefficient (95% CI)**		**Regression coefficient (95% CI)**	
**No adjustment**						
All subjects	**−0.221 (−0.369 to −0.074)**	**0.003**	−0.115 (−0.330 to 0.101)	0.297	**−0.818 (−1.196 to −0.440)**	**<0.001**
Prediabetes	0.036 (−0.366 to 0.437)	0.861	−0.094 (−0.548 to 0.36)	0.682	−0.438 (−1.566 to 0.689)	0.444
Diabetes	−0.409 (−0.894 to –0.077)	0.098	−0.344 (−1.091 to 0.403)	0.361	−1.086 (−2.197 to 0.026)	0.055
No diabetes/prediabetes	−0.116 (−0.312 to 0.081)	0.248	0.026 (−0.216 to 0.269)	0.831	**−0.512 (−0.986 to −0.039)**	**0.034**
**Adjusted for age and sex**						
All subjects	**−0.326 (−0.492 to −0.159)**	**<0.001**	−0.173 (−0.513 to 0.166)	0.316	**−1.213 (−1.659 to −0.768)**	**<0.001**
Prediabetes	−0.037 (−0.458 to 0.384)	0.862	−0.250 (−0.786 to 0.285)	0.355	−0.954 (−2.220 to 0.312)	0.139
Diabetes	−0.469 (−0.981 to 0.043)	0.072	−0.413 (−1.296 to 0.470)	0.353	**−1.366 (−2.593 to −0.138)**	**0.029**
No diabetes/prediabetes	**−0.268 (−0.481 to −0.056)**	**0.013**	−0.168 (−0.541 to 0.205)	0.377	**−0.961 (−1.501 to −0.421)**	**<0.001**
**Adjusted for age, sex and BMI**						
All subjects	**−0.239 (−0.412 to −0.066)**	**0.007**	0.024 (−0.351 to 0.399)	0.900	**−0.979 (−1.453 to −0.504)**	**<0.001**
Prediabetes	0.02 (−0.404 to 0.443)	0.927	−0.137 (−0.694 to 0.419)	0.625	−0.737 (−2.034 to 0.561)	0.264
Diabetes	−0.509 (−1.06 to 0.041)	0.070	−0.368 (−1.383 to 0.646)	0.471	**−1.459 (−2.759 to −0.159)**	**0.028**
No diabetes/prediabetes	−0.196 (−0.416 to 0.024)	0.080	0.020 (−0.392 to 0.432)	0.923	**−0.759 (−1.337 to −0.182)**	**0.010**
**Multivariable adjustment**^a^						
All subjects	−0.070 (−0.252 to 0.113)	0.455	0.189 (−0.204 to 0.582)	0.344	−0.526 (−1.061 to 0.010)	0.054
Prediabetes	0.176 (−0.263 to 0.615)	0.431	0.010 (−0.656 to 0.675)	0.977	0.573 (−0.925 to 2.071)	0.451
Diabetes	−0.173 (−0.800 to 0.453)	0.585	0.032 (−1.160 to 1.225)	0.957	−0.527 (−2.132 to 1.077)	0.516
No diabetes/prediabetes	−0.118 (−0.352 to 0.115)	0.319	0.030 (−0.414 to 0.475)	0.894	**−0.660 (−1.309 to −0.011)**	**0.046**

a Adjustment for age, sex, BMI, hypertension (yes/no), HDL cholesterol (continuous), LDL cholesterol (continuous), triglycerides (continuous), former myocardial infarction or stroke, smoking behaviour (active/former/never), alcohol consumption (never/moderate/high), physical activity (high/low), hsCRP, eGFR, statins, NSAID, beta-blockers, angiotensin converting enzyme inhibitors and angiotensin 1 receptor antagonists.
