# Supplementary material for: Dataset of the associations of aldosterone to renin ratio with MR-proANP and MR-proADM
Source: Data Brief. 2016 Aug 9;8:1395–9. doi: 10.1016/j.dib.2016.08.008 (PMC4995539; doi:10.1016/j.dib.2016.08.008)
Supplement: Supplementary file 1 — Supplementary material [file mmc1.pdf]

For submission to *Atherosclerosis*

Manuscript: Alterations of the renin-aldosterone system in type 2 diabetes: the KORA F4 study

Conflict of interest disclosure

We wish to confirm that there are no known conflicts of interest associated with this publication and there has been no significant financial support for this work that could have influenced its outcome.

We confirm that the manuscript has been read and approved by all named authors and that there are no other persons who satisfied the criteria for authorship but are not listed. We further confirm that the order of authors listed in the manuscript has been approved by all of us.

We confirm that we have given due consideration to the protection of intellectual property associated with this work and that there are no impediments to publication, including the timing of publication, with respect to intellectual property. In so doing we confirm that we have followed the regulations of our institutions concerning intellectual property.

We further confirm that any aspect of the work covered in this manuscript that has involved human patients has been conducted with the ethical approval of all relevant bodies and that such approvals are acknowledged within the manuscript.

We understand that the Corresponding Author is the sole contact for the Editorial process (including Editorial Manager and direct communications with the office). He/she is responsible for communicating with the other authors about progress, submissions of revisions and final approval of proofs. We confirm that we have provided a current, correct email address which is accessible by the Corresponding Author (cornelia.then@med.uni-muenchen.de).

Signed by all authors as follows:

Cornelia Then 14.10.2015

Marietta Rottenkolber

Andreas Lechner 15\10\15

Christa Meisinger

Margit Heier

Wolfgang Koenig

Annette Peters

Wolfgang Rathmann

Martin Bidlingmaier 21/10/15

Martin Reincke 14.10.15

Jochen Seissler 14-10-15

For submission to *Atherosclerosis*

Manuscript: Alterations of the renin-aldosterone system in type 2 diabetes: the KORA F4 study

Conflict of interest disclosure

We wish to confirm that there are no known conflicts of interest associated with this publication and there has been no significant financial support for this work that could have influenced its outcome.

We confirm that the manuscript has been read and approved by all named authors and that there are no other persons who satisfied the criteria for authorship but are not listed. We further confirm that the order of authors listed in the manuscript has been approved by all of us.

We confirm that we have given due consideration to the protection of intellectual property associated with this work and that there are no impediments to publication, including the timing of publication, with respect to intellectual property. In so doing we confirm that we have followed the regulations of our institutions concerning intellectual property.

We further confirm that any aspect of the work covered in this manuscript that has involved human patients has been conducted with the ethical approval of all relevant bodies and that such approvals are acknowledged within the manuscript.

We understand that the Corresponding Author is the sole contact for the Editorial process (including Editorial Manager and direct communications with the office). He/she is responsible for communicating with the other authors about progress, submissions of revisions and final approval of proofs. We confirm that we have provided a current, correct email address which is accessible by the Corresponding Author (cornelia.then@med.uni-muenchen.de).

Signed by all authors as follows:

Cornelia Then ______________________

Marietta Rottenkolber M. Rottenkolber

Andreas Lechner ______________________

Christa Meisinger ______________________

Margit Heier ______________________

Wolfgang Koenig ______________________

Annette Peters ______________________

Wolfgang Rathmann ______________________

Martin Bidlingmaier ______________________

Martin Reincke ______________________

Jochen Seissler ______________________

For submission to *Atherosclerosis*

Manuscript: Alterations of the renin-aldosterone system in type 2 diabetes: the KORA F4 study

Conflict of interest disclosure

We wish to confirm that there are no known conflicts of interest associated with this publication and there has been no significant financial support for this work that could have influenced its outcome.

We confirm that the manuscript has been read and approved by all named authors and that there are no other persons who satisfied the criteria for authorship but are not listed. We further confirm that the order of authors listed in the manuscript has been approved by all of us.

We confirm that we have given due consideration to the protection of intellectual property associated with this work and that there are no impediments to publication, including the timing of publication, with respect to intellectual property. In so doing we confirm that we have followed the regulations of our institutions concerning intellectual property.

We further confirm that any aspect of the work covered in this manuscript that has involved human patients has been conducted with the ethical approval of all relevant bodies and that such approvals are acknowledged within the manuscript.

We understand that the Corresponding Author is the sole contact for the Editorial process (including Editorial Manager and direct communications with the office). He/she is responsible for communicating with the other authors about progress, submissions of revisions and final approval of proofs. We confirm that we have provided a current, correct email address which is accessible by the Corresponding Author (cornelia.then@med.uni-muenchen.de).

Signed by all authors as follows:

Cornelia Then ______________________

Marietta Rottenkolber ______________________

Andreas Lechner ______________________

Christa Meisinger Meisinger C. October 13, 2015

Margit Heier ______________________

Wolfgang Koenig ______________________

Annette Peters ______________________

Wolfgang Rathmann ______________________

Martin Bidlingmaier ______________________

Martin Reincke ______________________

Jochen Seissler ______________________

For submission to *Atherosclerosis*

Manuscript: Alterations of the renin-aldosterone system in type 2 diabetes: the KORA F4 study

Conflict of interest disclosure

We wish to confirm that there are no known conflicts of interest associated with this publication and there has been no significant financial support for this work that could have influenced its outcome.

We confirm that the manuscript has been read and approved by all named authors and that there are no other persons who satisfied the criteria for authorship but are not listed. We further confirm that the order of authors listed in the manuscript has been approved by all of us.

We confirm that we have given due consideration to the protection of intellectual property associated with this work and that there are no impediments to publication, including the timing of publication, with respect to intellectual property. In so doing we confirm that we have followed the regulations of our institutions concerning intellectual property.

We further confirm that any aspect of the work covered in this manuscript that has involved human patients has been conducted with the ethical approval of all relevant bodies and that such approvals are acknowledged within the manuscript.

We understand that the Corresponding Author is the sole contact for the Editorial process (including Editorial Manager and direct communications with the office). He/she is responsible for communicating with the other authors about progress, submissions of revisions and final approval of proofs. We confirm that we have provided a current, correct email address which is accessible by the Corresponding Author (cornelia.then@med.uni-muenchen.de).

Signed by all authors as follows:

Cornelia Then __________

Marietta Rottenkolber __________

Andreas Lechner __________

Christa Meisinger __________

Margit Heier M. Heier 13- Oct - 2015

Wolfgang Koenig __________

Annette Peters __________

Wolfgang Rathmann __________

Martin Bidlingmaier __________

Martin Reincke __________

Jochen Seissler __________

For submission to *Atherosclerosis*

Manuscript: Alterations of the renin-aldosterone system in type 2 diabetes: the KORA F4 study

Conflict of interest disclosure

We wish to confirm that there are no known conflicts of interest associated with this publication and there has been no significant financial support for this work that could have influenced its outcome.

We confirm that the manuscript has been read and approved by all named authors and that there are no other persons who satisfied the criteria for authorship but are not listed. We further confirm that the order of authors listed in the manuscript has been approved by all of us.

We confirm that we have given due consideration to the protection of intellectual property associated with this work and that there are no impediments to publication, including the timing of publication, with respect to intellectual property. In so doing we confirm that we have followed the regulations of our institutions concerning intellectual property.

We further confirm that any aspect of the work covered in this manuscript that has involved human patients has been conducted with the ethical approval of all relevant bodies and that such approvals are acknowledged within the manuscript.

We understand that the Corresponding Author is the sole contact for the Editorial process (including Editorial Manager and direct communications with the office). He/she is responsible for communicating with the other authors about progress, submissions of revisions and final approval of proofs. We confirm that we have provided a current, correct email address which is accessible by the Corresponding Author (cornelia.then@med.uni-muenchen.de).

Signed by all authors as follows:

Cornelia Then ____________________

Marietta Rottenkolber ____________________

Andreas Lechner ____________________

Christa Meisinger ____________________

Margit Heier ____________________

Wolfgang Koenig W. Koenig 27.10.15

Annette Peters ____________________

Wolfgang Rathmann ____________________

Martin Bidlingmaier ____________________

Martin Reincke ____________________

Jochen Seissler ____________________

For submission to *Atherosclerosis*

Manuscript: Alterations of the renin-aldosterone system in type 2 diabetes: the KORA F4 study

Conflict of interest disclosure

We wish to confirm that there are no known conflicts of interest associated with this publication and there has been no significant financial support for this work that could have influenced its outcome.

We confirm that the manuscript has been read and approved by all named authors and that there are no other persons who satisfied the criteria for authorship but are not listed. We further confirm that the order of authors listed in the manuscript has been approved by all of us.

We confirm that we have given due consideration to the protection of intellectual property associated with this work and that there are no impediments to publication, including the timing of publication, with respect to intellectual property. In so doing we confirm that we have followed the regulations of our institutions concerning intellectual property.

We further confirm that any aspect of the work covered in this manuscript that has involved human patients has been conducted with the ethical approval of all relevant bodies and that such approvals are acknowledged within the manuscript.

We understand that the Corresponding Author is the sole contact for the Editorial process (including Editorial Manager and direct communications with the office). He/she is responsible for communicating with the other authors about progress, submissions of revisions and final approval of proofs. We confirm that we have provided a current, correct email address which is accessible by the Corresponding Author (cornelia.then@med.uni-muenchen.de).

Signed by all authors as follows:

Cornelia Then ______________________

Marietta Rottenkolber ______________________

Andreas Lechner ______________________

Christa Meisinger ______________________

Margit Heier ______________________

Wolfgang Koenig ______________________

Annette Peters [illegible] 13.10.15

Wolfgang Rathmann ______________________

Martin Bidlingmaier ______________________

Martin Reincke ______________________

Jochen Seissler ______________________

For submission to *Atherosclerosis*

Manuscript: Alterations of the renin-aldosterone system in type 2 diabetes: the KORA F4 study

Conflict of interest disclosure

We wish to confirm that there are no known conflicts of interest associated with this publication and there has been no significant financial support for this work that could have influenced its outcome.

We confirm that the manuscript has been read and approved by all named authors and that there are no other persons who satisfied the criteria for authorship but are not listed. We further confirm that the order of authors listed in the manuscript has been approved by all of us.

We confirm that we have given due consideration to the protection of intellectual property associated with this work and that there are no impediments to publication, including the timing of publication, with respect to intellectual property. In so doing we confirm that we have followed the regulations of our institutions concerning intellectual property.

We further confirm that any aspect of the work covered in this manuscript that has involved human patients has been conducted with the ethical approval of all relevant bodies and that such approvals are acknowledged within the manuscript.

We understand that the Corresponding Author is the sole contact for the Editorial process (including Editorial Manager and direct communications with the office). He/she is responsible for communicating with the other authors about progress, submissions of revisions and final approval of proofs. We confirm that we have provided a current, correct email address which is accessible by the Corresponding Author (cornelia.then@med.uni-muenchen.de).

Signed by all authors as follows:

Cornelia Then ____________________

Marietta Rottenkolber ____________________

Andreas Lechner ____________________

Christa Meisinger ____________________

Margit Heier ____________________

Wolfgang Koenig ____________________

Annette Peters ____________________

Wolfgang Rathmann ____________________

Martin Bidlingmaier ____________________

Martin Reincke ____________________

Jochen Seissler ____________________
